# Metabolism of l-arabinose in plants

**DOI:** 10.1007/s10265-016-0834-z

**Published:** 2016-05-24

**Authors:** Toshihisa Kotake, Yukiko Yamanashi, Chiemi Imaizumi, Yoichi Tsumuraya

**Affiliations:** Division of Life Science, Graduate School of Science and Engineering, Saitama University, 255 Shimo-okubo, Sakura-ku, Saitama, 338-8570 Japan

**Keywords:** l-Arabinose, Cell wall polysaccharide, Cytosol, Golgi apparatus, Nucleotide sugar

## Abstract

l-Arabinose (l-Ara) is a plant-specific sugar accounting for 5–10 % of cell wall saccharides in Arabidopsis (*Arabidopsis thaliana*) and rice (*Oryza sativa*). l-Ara occurs in pectic arabinan, rhamnogalacturonan II, arabinoxylan, arabinogalactan-protein (AGP), and extensin in the cell walls, as well as in glycosylated signaling peptides like CLAVATA3 and small glycoconjugates such as quercetin 3-*O*-arabinoside. This review focuses on recent advances towards understanding the generation of l-Ara and the metabolism of l-Ara-containing molecules in plants.

**Electronic supplementary material:**

The online version of this article (doi:10.1007/s10265-016-0834-z) contains supplementary material, which is available to authorized users.

## Introduction

**Fig. 1:**
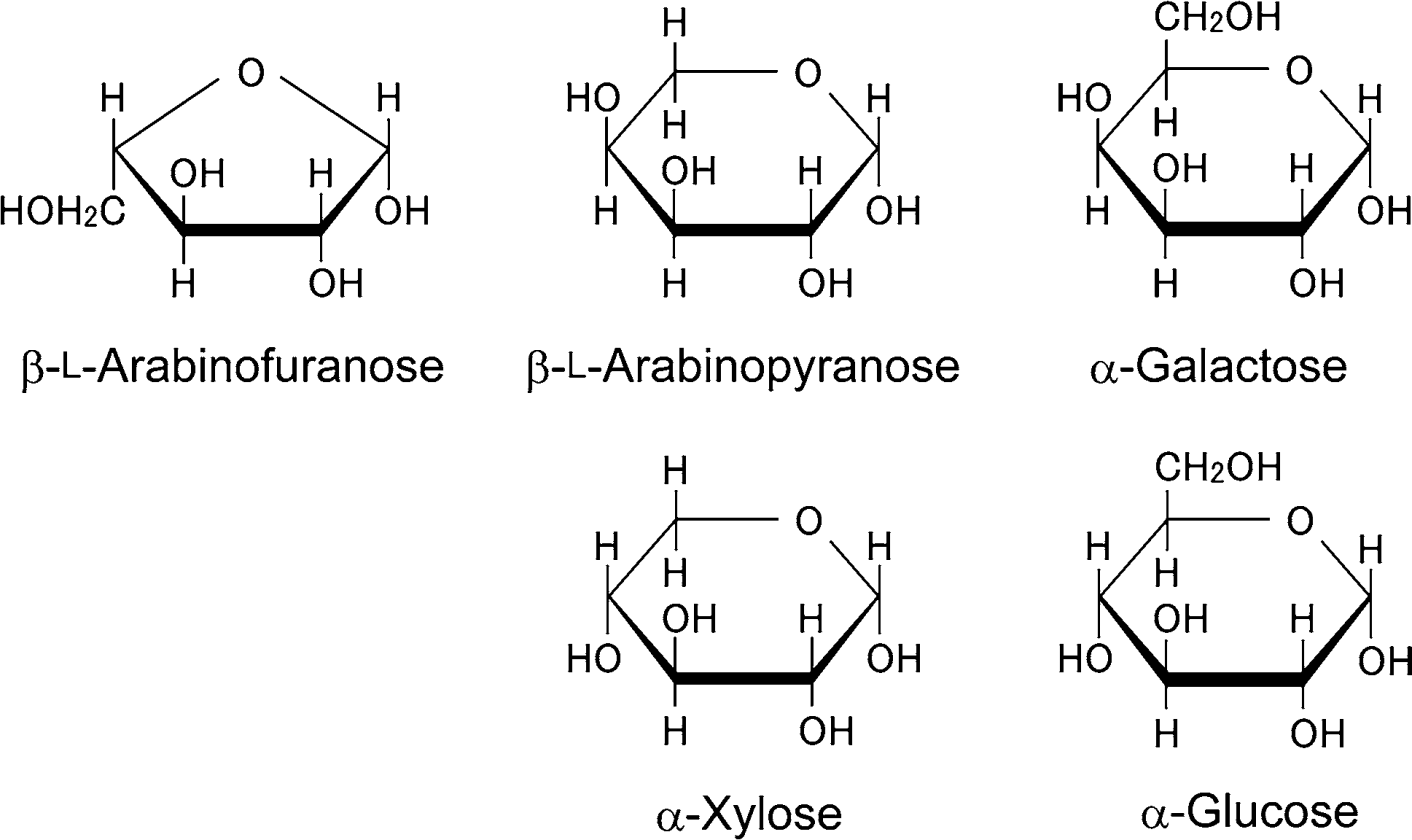
Structure of β-l-Ara*f* and β-l-Ara*p*. Sugars are drawn in the Haworth projection. The furanose β-l-Ara*f* has the shape of a pentagon, whereas the pyranose β-l-Ara*p* forms a hexagon. The structure of β-l-Ara*p* is similar to that of α-Gal and the C-4 epimer of α-Xyl. l-Ara and Xyl are pentoses, whereas Gal and Glc are hexoses

l-Arabinose (l-Ara) is a plant saccharide that is not found in animals. Like xylose (Xyl—we omit the D-prefix of sugars belonging to the d-series), l-Ara is a pentose comprising five carbons, not six carbon containing hexose like glucose (Glc) and galactose (Gal) (Fig. 1). Although its content in the cell walls varies depending on the plant species, l-Ara can be considered a major sugar. It accounts for 5–10 % of cell wall sugar, for instance, in Arabidopsis (*Arabidopsis thaliana*) and rice (*Oryza sativa*) (Konishi et al. 2011; Zablackis et al. 1995). The fact that l-Ara is widely distributed not only in land plants including liverworts and mosses but also found in several Chlorophycean and Charophycean green algae suggests that the metabolic pathway for the synthesis of l-Ara was acquired early by primitive plants (Domozych et al. 2009, 2012; Konno et al. 2010; Lee et al. 2005; Popper and Fry 2003; Roberts et al. 2012; Thomas 1977). The broad range of l-Ara-containing molecules seen in land plants today is likely due to subsequent diversification of the use of l-Ara during plant evolution.

l-Ara may be useful as a natural pharmaceutical. Monomeric l-Ara inhibits intestinal maltase and sucrase (α-glucosidase hydrolyzing sucrose) activities in vitro (Seri et al. 1996). In rats, dietary sucrose increases the insulin level in blood and triacylglycerol levels in blood plasma and the liver, but feeding l-Ara together with sucrose can significantly reduce the increase in these levels (Osaki et al. 2001; Seri et al. 1996). Recently, the effect of l-Ara on controlling insulin and blood-Glc levels was also observed in humans (Kaats et al. 2011). While its effect in humans is still controversial (Halschou-Jensen et al. 2015), the use of l-Ara for these purposes is receiving attention and becoming more wide-spread.

Several excellent reviews have surveyed nucleotide sugar synthesis and sugar metabolism in land plants (Bar-Peled and O’Neill 2011; Bar-Peled et al. 2012; Lagaert et al. 2014; Reiter 2008; Reiter and Vanzin 2001; Seifert 2004). Here we concentrate on recent progress in our understanding of the generation of l-Ara and the synthesis and degradation of l-Ara-containing molecules in land plants.

## l-Ara-containing molecules in plants

l-Ara has two ring forms, called l-arabinopyranose (l-Ara*p*, sugars other than l-Ara are in pyranose form unless stated otherwise) and l-arabinofuranose (l-Ara*f*), respectively (Fig. 1). Free l-Ara exists as l-Ara*p* in solution because the pyranose form is more stable than the furanose, but among cell wall polysaccharides and glycoproteins/proteoglycans, l-Ara*f* residues outnumber the l-Ara*p* residues. Representative l-Ara-containing molecules in plants are listed in Table 1.

**Table 1:** l-Ara-containing molecules in plants

Molecule	Plant species^a^	l-Ara residue(s)	References
Cell wall polysaccharide			
RG-I α-1,3:1,5-arabinan	*Beta vulgaris*	α-l-Ara*f*	Levigne et al. (2004); Westphal et al. (2010)
RG-I type I AG	*Glycine max*	α-l-Ara*f*	Nakamura et al. (2001)
RG-II	Arabidopsis	β-l-Ara*f*, α-l-Ara*p*	O’Neill et al. (2001)
Arabinoxylan	*Triticum aestivum*	α-l-Ara*f*	Virkki et al. (2008); Anders et al. (2012)
Xyloglucan	*Solanum tuberosum*	α-l-Ara*f*	Vincken et al. (1996)
Cell wall glycoprotein/proteoglycan			
AGP (type II AG)	*Triticum aestivum*	α-l-Ara*f*, β-l-Ara*p*	Tryfona et al. (2010)
Extensin	*Nicotiana tabacum*	α-l-Ara*f*; β-l-Ara*f*	Akiyama et al. (1980); McNeil et al. (1984)
Signaling peptide			
CLE peptide	Arabidopsis	β-l-Ara*f*	Ohyama et al. (2009)
Flavonoid			
Myricetin glycoside	*Calycolpus warszewiczianus*	α-l-Ara*f*	Torres-Mendoza et al. (2006)
Quercetin glycoside	*Anthyllis hermanniae*	α-l-Ara*p*	Halabalaki et al. (2011)

^a^A representative plant species is shown

**Fig. 2:**
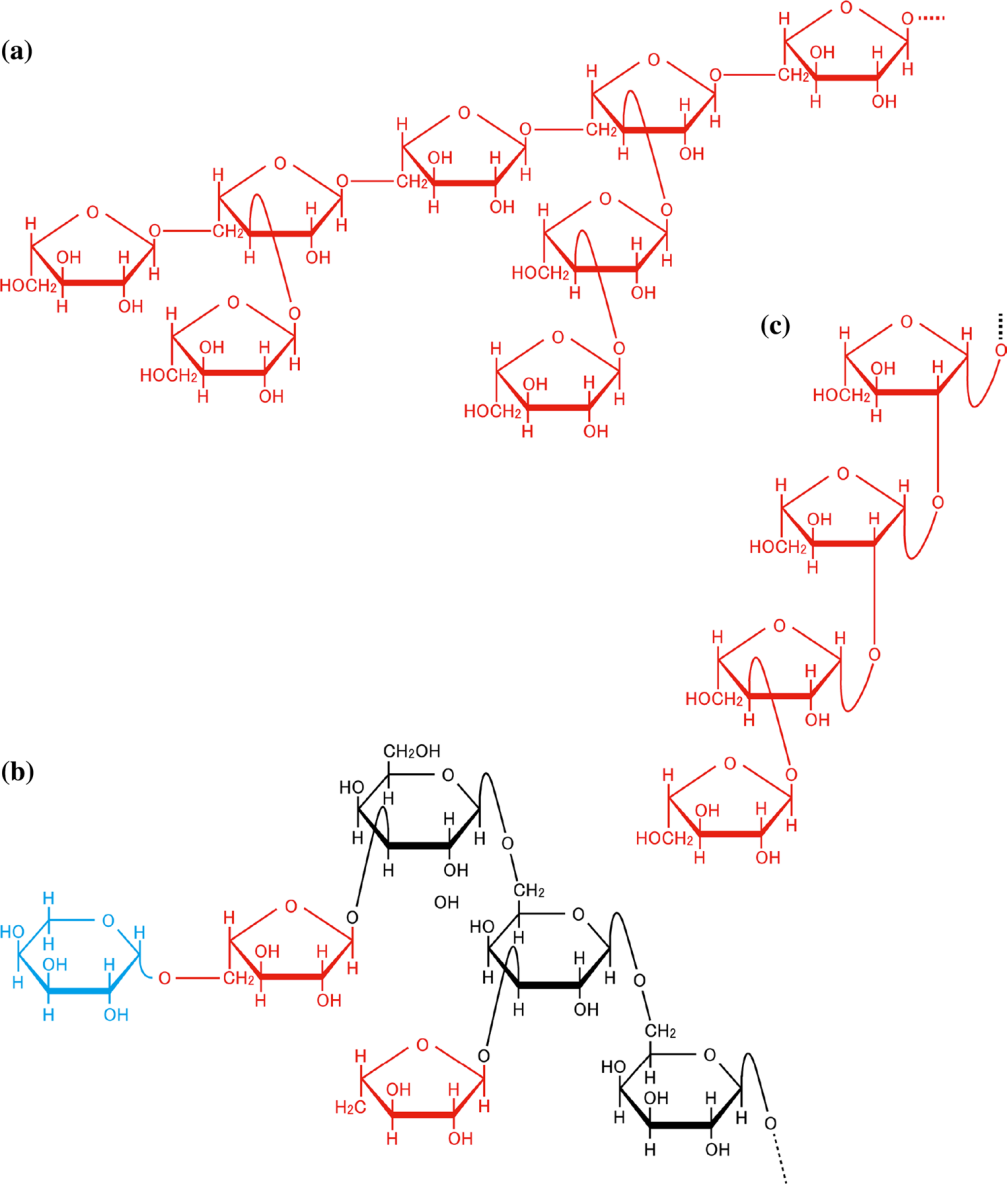
Structure of l-Ara-containing molecules. A few representative l-Ara-containing molecules, **a** pectic α-1,3:1,5-arabinan, **b** side chain of type II AG, and **c** arabinooligosaccharide of extensin. l-Ara*f* residues are *red*, the only l-Ara*p* residue is shown in *blue*

Pectin is a complex molecule with many different domains, including homogalacturonan, rhamnogalacturonan I (RG-I), and rhamnogalacturonan II (RG-II) (Mohnen 2008; Tan et al. 2013; Willats et al. 2001). Pectic RG-I is a domain to which α-1,3:1,5-arabinan (pectic arabinan) and type I arabinogalactan (AG) are attached (Table 1, Fig. 2a) (Mohnen 2008). The pectic arabinan consists of α-l-Ara*f* residues and is a major l-Ara-containing molecule in the cell walls in many plants (Levigne et al. 2004). ARABINAN DEFICIENT 1 (ARAD1) and ARAD2 are glycosyltransferases associated with the synthesis of pectic arabinan (Harholt et al. 2012). Based on amino acid sequence and structural similarity, ARADs are categorized into glycosyltransferase family (GTF) 47 (Campbell et al. 1997; Coutinho et al. 2003) (Table 2). The importance of pectic arabinan in the regulation of stomata opening was demonstrated in a study using endo-α-1,5-arabinanase specifically degrading α-1,5-arabinan main chains (Jones et al. 2003). α-l-Ara*f* residues also exist in type I AG, another type of RG-I side chain, where they appear as non-reducing terminal residues (Nakamura et al. 2001) (Table 1). RG-II is the most complicated domain comprising more than ten types of sugars and includes β-l-Ara*f* and α-l-Ara*p* residues (Bar-Peled et al. 2012; O’Neill et al. 2001) (Table 1).

**Table 2:** Glycosyltransferases involved in the synthesis of l-Ara residues

Enzyme name	GTF	Product residue^a^	Molecule	References
Cell wall polysaccharide				
ARAD	47	**α-** **l** **-Araf**-1,5-α-l-Ara*f*	RG-I arabinan	Harholt et al. (2006)
XAT	61	**α-** **l** **-Araf**-1,3-β-Xyl	Arabinoxylan	Anders et al. (2012)
XST	47	**α-** **l** **-Araf**-1,2-α-Xyl	Xyloglucan	Schultink et al. (2013)
Cell wall glycoprotein/proteoglycan				
ArapT	n.d.^b^	**β-** **l** **-Arap**-1,3-α-l-Ara*f*	Type II AG	Ishii et al. (2005)
Signaling peptide				
HPAT	95	**β-** **l** **-Araf**-*O*-Hyp	CLV3 peptide	Ogawa-Ohnishi et al. (2013)
RRA3a	77	**β-** **l** **-Araf**-1,2-β-l-Ara*f*	CLV3 peptide	Xu et al. (2015)
Flavonol				
UGT78D3	1	**α-** **l** **-Arap**-*O*-quercetin	Quercetin 3-*O*-l-arabinoside	Yonekura-Sakakibara et al. (2008)

^a^Residue synthesized by the enzyme is bold

^b^Not determined, because the enzyme has not been cloned

Arabinogalactan-proteins (AGPs) constitute a family of plant extracellular proteoglycans with a large carbohydrate moiety rich in l-Ara and Gal. In order to distinguish it from the type I AG of pectin, the glycan of AGP is called type II AG. The basic structure of type I AG is β-1,4-galactan, whereas that of type II AG is β-1,3:1,6-galactan (main chain, β-1,3-galactan; side chain, β-1,6-galactan) (Shimoda et al. 2014; Tan et al. 2004, 2010; Tsumuraya et al. 1988). In AGP, α-l-Ara*f* residues exist as non-reducing terminal residues of type II AG. AGP sometimes has continuous α-l-Ara*f* residues linked through α-1,5-linkages, thus resembling pectic arabinan (Tan et al. 2004; Tryfona et al. 2012). However, it is still unknown whether ARADs also participate in the synthesis of this structure in AGP. In wheat AGP, the α-l-Ara*f* residues are further substituted with β-l-Ara*p* residues (Tryfona et al. 2010) (Table 1; Fig. 2b). The activity of glycosyltransferase (ArapT) catalyzing the transfer of β-l-Ara*p* from UDP-l-Ara*p* onto α-l-Ara*f* residues was detected in the microsomal fraction of mung bean seedlings (Ishii et al. 2005), but the gene encoding this glycosyltransferase has not been identified (Table 2).

In the vegetative tissues of grasses, including rice (*Oryza sativa*) and wheat (*Triticum aestivum*), instead of pectic arabinan, arabinoxylan is a major l-Ara-containing molecule in the cell walls (Table 1). The α-l-Ara*f* residues are attached to the β-1,4-xylan main chain through α-1,2- and/or α-1,3-linkages. The α-1,3-l-Ara*f* residues of arabinoxylan are formed by xylan arabinofuranosyltransferase (XAT), which belongs to GTF 61 (Anders et al. 2012). Interestingly, the α-l-Ara*f* residues can be further substituted with ferulic acid, which forms cross-links between arabinoxylans (Grabber et al. 1995; Saulnier et al. 1999). This cross-link formation is physiologically important, as it is regulated by environmental signals including light and osmotic stress and affects cell wall extensibility, thereby controlling growth and development (Parvez et al. 1997; Tan et al. 1992; Wakabayashi et al. 1997, 2015).

Xyloglucan is a major hemicellulosic polysaccharide in many dicotyledonous plants. This polysaccharide usually consists of β-Glc, α-Xyl, β-Gal, and α-l-fucose (α-l-Fuc), but in several plants such as potato and olive, the β-Gal residues are replaced by α-l-Ara*f* residues (Table 1) (Jia et al. 2003; Vierhuis et al. 2001; Vincken et al. 1996; York et al. 1996). The glycosyltransferases catalyzing the transfer of α-l-Ara*f* residues onto the xylosyl (Xyl) residues, xyloglucan S-side chain transferases (XSTs), have been identified. XSTs are members of GTF 47, which also includes Xyloglucan l-side chain galactosylTransferase 2 (XLT2) and MURUS3 catalyzing the transfer of β-Gal residues onto the Xyl residues (Schultink et al. 2013).

Extensins form a class of cell wall glycoproteins with Hyp-rich core-protein and contain arabino-oligosaccharides consisting of α-l-Ara*f* and β-l-Ara*f* residues (Kieliszewski et al. 1995; Lamport et al. 1973; McNeill et al. 1984) (Fig. 2c; Table 1). Surprisingly, a glycoprotein from *Chlamydomonas reinhardtii* appears to have similar arabinan chains, that is, the proximal two residues linked to Hyp, β-l-Ara*f*1→2β-l-Ara*f*1→Hyp, are identical to those of extensin (Bollig et al. 2007). This fact suggests that some of Chlorophycean green algae and land plants share the basic mechanism for the synthesis of this arabino-oligosaccharides and points to the possibility that l-arabinofuranosyltransferases and the metabolic pathway for UDP-l-Ara*f* may be conserved.

Extensin-type arabino-oligosaccharides are also attached to glycosylated signaling peptides, the CLAVATA3 (CLV3)/Endosperm surrounding region-related (CLE) peptides (Ohyama et al. 2009; Okamoto et al. 2013; Xu et al. 2015). Using synthetic peptides with or without β-l-Ara*f*1→2β-l-Ara*f*1→2β-l-Ara*f* chain, it has been demonstrated that the arabino-oligosaccharide is necessary for the proper function of CLV3 as a signaling molecule (Ohyama et al. 2009). The transfer of the first β-l-Ara*f* residue onto Hyp is catalyzed by Hyp *O*-arabinosyltransferases (HPAT) classified into GTF 95 (Table 2). Indeed, loss of function mutations of *HPAT* genes causes pleiotropic phenotypes in Arabidopsis (Ogawa-Ohnishi et al. 2013). The importance of this arabino-oligosaccharide is further demonstrated by the existence of a tomato inflorescence branching mutant with extra flower and fruit organs, which has defects in a gene encoding GTF77 β-l-arabinofuranosyltransferase that synthesizes the β-l-Ara*f* residues, and can be rescued by treatment with l-arabinofuranosylated CLV3 peptide (Xu et al. 2015).

Most l-Ara-containing polysaccharides, proteoglycans, glycoproteins, and secreted peptides are synthesized in the Golgi apparatus, but small l-Ara-containing glycoconjugates are synthesized in the cytosol. Flavonoids are good examples of small glycoconjugates with l-Ara*f* and l-Ara*p* residues (Table 1). In Arabidopsis, a number of l-arabinopyranosylated flavonols have been found (Tueber and Herrmann 1978; Yonekura-Sakakibara et al. 2008). By transcriptome co-expression network analysis using public databases, an l-arabinosyltransferase, UGT78D3, was identified. This enzyme participates in the synthesis of quercetin 3-*O*-l-arabinoside in Arabidopsis (Yonekura-Sakakibara et al. 2008) (Table 2). Other l-arabinopyranosylated small glycoconjugates were also found. Floratheasaponin in the tea (*Camellia sinensis*) plant has an α-l-Ara*p* residue in its carbohydrate moiety (Yoshikawa et al. 2005).

## Generation of l**-**Ara

**Fig. 3:**
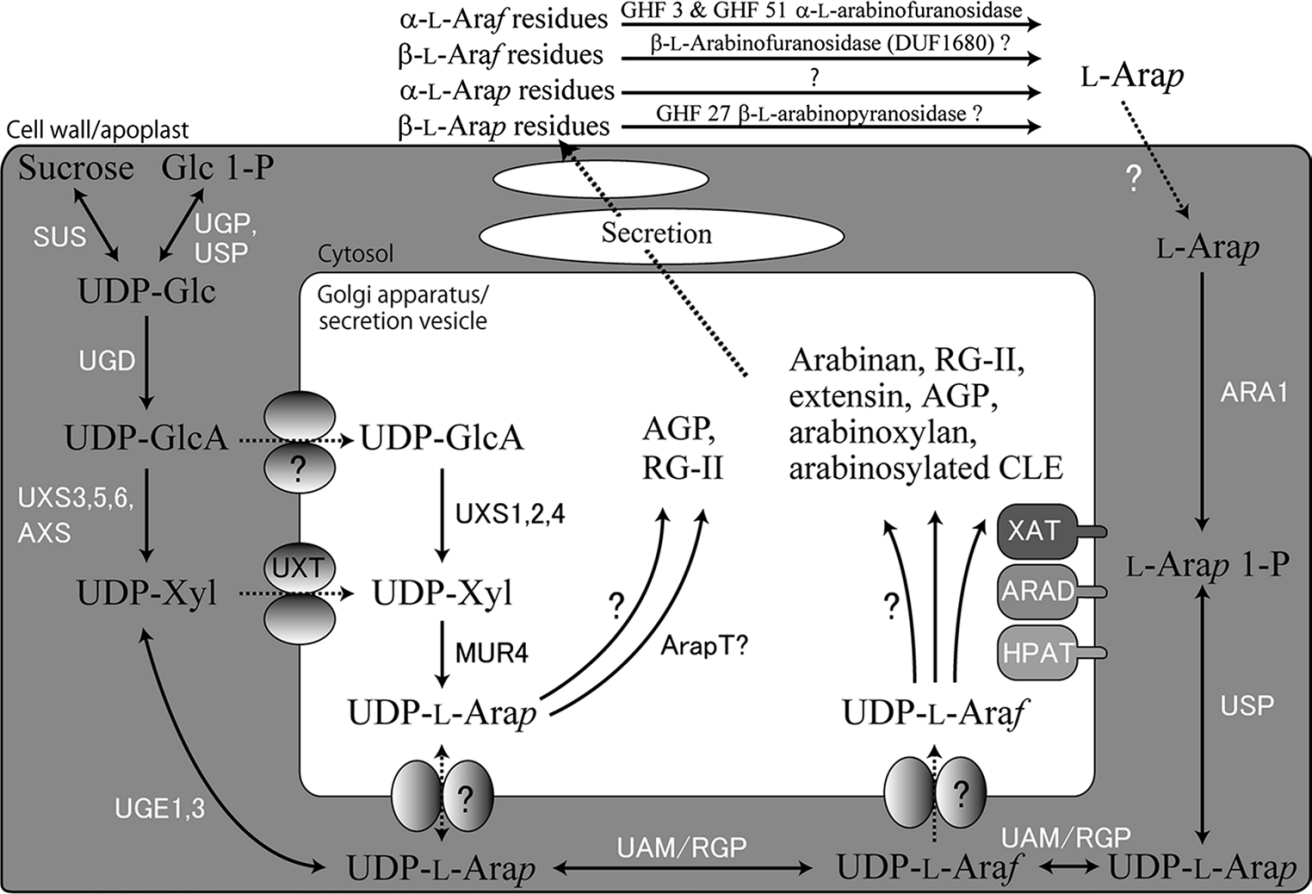
Synthesis and degradation of l-Ara-containing molecules. UDP-l-Ara*p* is synthesized in the de novo pathway in the Golgi apparatus and cytosol, which is shown in the *left side*. UDP-l-Ara*p* is further converted to UDP-l-Ara*f* in the cytosol. UDP-l-Ara*f* and UDP-l-Ara*p* serve as donor substrates in the synthesis of l-Ara-containing molecules. l-Ara-containing molecules undergo degradation by various glycoside hydrolases in the cell walls. The released l-Ara is recycled to UDP-l-Ara*p* in the salvage pathway shown in the *right side*. The figure combines several reactions routes found in various plant species and is not to be understood to depict pathways found in any one particular plant. The enzymes in the de novo and salvage pathway are listed in Table 3

l-Ara is synthesized as a form of UDP-l-Ara*p* from UDP-Xyl by UDP-Xyl 4-epimerases (UXEs) through C-4 epimerization of UDP-Xyl (Fig. 3). This is the only reaction route known to generate l-Ara in plants so far. It is also possible to synthesize UDP-l-Ara*p* from UDP-galacturonic acid (UDP-GalA) through C-6 decarboxylation. However, no UDP-GalA decarboxylase forming UDP-l-Ara*p* from UDP-GalA has been found in plants so far, although UDP-glucuronic acid (UDP-GlcA) decarboxylase (other name, UDP-Xyl synthase, UXS) forming UDP-Xyl from UDP-GlcA exists in many eukaryotes, including plants (Bar-Peled et al. 2001; Harper and Bar-Peled 2002).

The reaction synthesizing UDP-l-Ara*p* is part of the de novo pathway for UDP-sugars (Fig. 3). The enzymes constituting the de novo pathway are listed in Table 3. In the de novo pathway, UDP-Glc, the starting substrate of this pathway, is first synthesized from sucrose and UDP by sucrose synthase (SUS) (Baud et al. 2004; Cardini et al. 1955) or from Glc 1-phosphate (Glc 1-P) and UTP by UDP-Glc pyrophosphorylase (UGP) (Meng et al. 2009; Park et al. 2010) or UDP-sugar pyrophosphorylase (USP) (Kotake et al. 2004, 2007; Litterer et al. 2006). UDP-Glc undergoes C-6 oxidation, which turns it into UDP-GlcA by UDP-Glc dehydrogenase (UGD) (Reboul et al. 2011), and then undergoes C-6 decarboxylation to form UDP-Xyl by UXS. Plants have Golgi-localized and cytosolic UXSs, implying that the pathway is dual. In Arabidopsis, UXS1, 2, and 4 with a transmembrane domain at the N-terminus produce UDP-Xyl as Golgi-localized enzymes, whereas UXS3, 5, and 6 catalyze the same reaction in the cytosol. Other enzymes, UDP-apiose/UDP-Xyl synthases (AXSs) also participate in this reaction in the cytosol (Mølhøj et al. 2003). Following l-Ara forming reaction, C-4 epimerization of UDP-Xyl to form UDP-l-Ara*p*, also occurs both in the Golgi apparatus and cytosol. In the Golgi apparatus, the formation of UDP-l-Ara*p* is catalyzed by a Golgi-localized UXE, MURUS4 (MUR4) (Burget and Reiter 1999; Burget et al. 2003), but in the cytosol, it is catalyzed by bifunctional UGE1 and UGE3 in Arabidopsis (Fig. 3). Among five UGEs, only UGE1 and UGE3 possess UXE activity beside UDP-Glc 4-epimerase activity in Arabidopsis (Kotake et al. 2009). An Arabidopsis *mur4* mutant shows a 50 % reduction in cell wall l-Ara, but a *uge1 uge3* double mutant has normal cell walls (Burget and Reiter 1999; Rösti et al. 2007). These observations suggest that, at least in Arabidopsis, the main reaction to generate UDP-l-Ara*p* occurs in the Golgi apparatus. The role of the cytosolic pathway may be clarified in the future via studies on *mur4 uge1 uge3* triple mutants.

**Table 3:** Enzymes in the de novo and salvage pathways for UDP- l-Ara*p* and UDP-l-Ara*f*

Enzyme	Abbreviation^a^	Catalyzing reaction(s)
Enzymes in the de novo pathway		
Sucrose synthase	SUS	Sucrose + UDP ↔ UDP-Glc + fructose
UDP-Glc pyrophosphorylase	UGP	UTP + Glc 1-P ↔ UDP-Glc + PPi
UDP-Glc dehydrogenase	UGD	UDP-Glc → UDP-GlcA
UDP-GlcA decarboxylase	UXS	UDP-GlcA → UDP-Xyl
Golgi-localized UDP-Xyl 4-epimerase	MUR4	UDP-Xyl ↔ UDP-l-Ara*p*
UDP-Glc 4-epimerase	UGE	UDP-Glc ↔ UDP-Gal
Bifunctional UDP-Glc 4-epimerase	UGE1, UGE3	UDP-Glc ↔ UDP-Gal, UDP-Xyl ↔ UDP-l-Ara*p*
UDP-l-Ara*p* mutase	UAM/RGP	UDP- l-Ara*p* ↔ UDP-l-Ara*f*
Enzymes in the salvage pathway		
l-Arabinokinase	ARA1	l-Ara*p* + ATP → l-Ara*p* 1-P + ADP
UDP-sugar pyrophosphorylase^b^	USP	UTP + monosaccharide 1-P ↔ UDP-sugar + PPi

^a^The abbreviations for enzymes in Arabidopsis are shown

^b^This enzyme can also form UDP-Glc in the de novo pathway

## Conversion from UDP-**l-**Ara*p* to UDP-**l-**Ara*f*

The metabolism of UDP-l-Ara*p* turned out to be more complicated than expected, when the subsequent conversion to UDP-l-Ara*f* was investigated. The interconversion between UDP-l-Ara*p* and UDP-l-Ara*f* is catalyzed by a cytosolic enzyme, UDP-l-Ara*p* mutase/reversibly glycosylated protein (UAM/RGP, Drakakaki et al. 2006; Konishi et al. 2007; Konishi et al. 2011). No Golgi-localized enzyme catalyzing this reaction has been found so far. It thus looks very much as if the main reaction to convert UDP-Xyl to UDP-l-Ara*p* occurs in the Golgi apparatus, but the following conversion of UDP-l-Ara*p* to UDP-l-Ara*f* takes place in the cytosol. For this to work, two specific nucleotide sugar transporters (NSTs) would seem to be necessary: one transporter exporting UDP-l-Ara*p* from the Golgi apparatus and one importing UDP-l-Ara*f* into the Golgi apparatus. To efficiently incorporate synthesized UDP-l-Ara*f* back into the Golgi apparatus, the mutase reaction may occur around the Golgi apparatus in the cytosol. Supporting this view, a recent proteomics analysis has revealed that one of the UAM/RGPs, RGP4, is associated with the Golgi apparatus in Arabidopsis (Nikolovski et al. 2012). NSTs are a family of proteins including 40 members in Arabidopsis, which are categorized into six subgroups (Rautengarten et al. 2014). To date, plant-specific NSTs for GDP-sugars, UDP-GalA/UDP-l-rhamnose, and UDP-Xyl are known (Baldwin et al. 2001; Ebert et al. 2015; Rautengarten et al. 2014), but those for UDP-l-Ara*p* and UDP-l-Ara*f* remain to be identified. While l-Ara mainly exists as a form of l-Ara*f* residues in the cell walls, the level of UDP-l-Ara*f* is lower than that of UDP-l-Ara*p* in plant tissues (Ito et al. 2014; Pabst et al. 2010). It may be derived from the instability of UDP-l-Ara*f* under the experimental condition. It is also conceivable that UDP-l-Ara*f* imported by the transporter into the Golgi apparatus is immediately consumed by l-arabinofuranosyltransferases.

## Origin of UDP-Xyl 4-epimerase

As described above, l-Ara is synthesized as a form of UDP-l-Ara*p* by Golgi-localized MUR4 and cytosolic UGEs in land plants. These enzymes have similar amino acid sequences and are all categorized into the Rossmann fold superfamily (Rao and Rossman 1973), which also includes other UDP-sugar metabolizing enzymes: AXS, UXS, UDP-GlcA 4-epimerase, and UDP-l-rhamnose synthase (Diet et al. 2006; Gu and Bar-Peled 2004; Harper and Bar-Peled 2002; Mølhøj et al. 2003). In Arabidopsis, both of MUR4 and the bifunctional UGEs possess UXE activity, but the origin of these proteins seems different. It has thus been suggested that the UXE MUR4 is likely older than the bifunctional UGE.

**Fig. 4:**
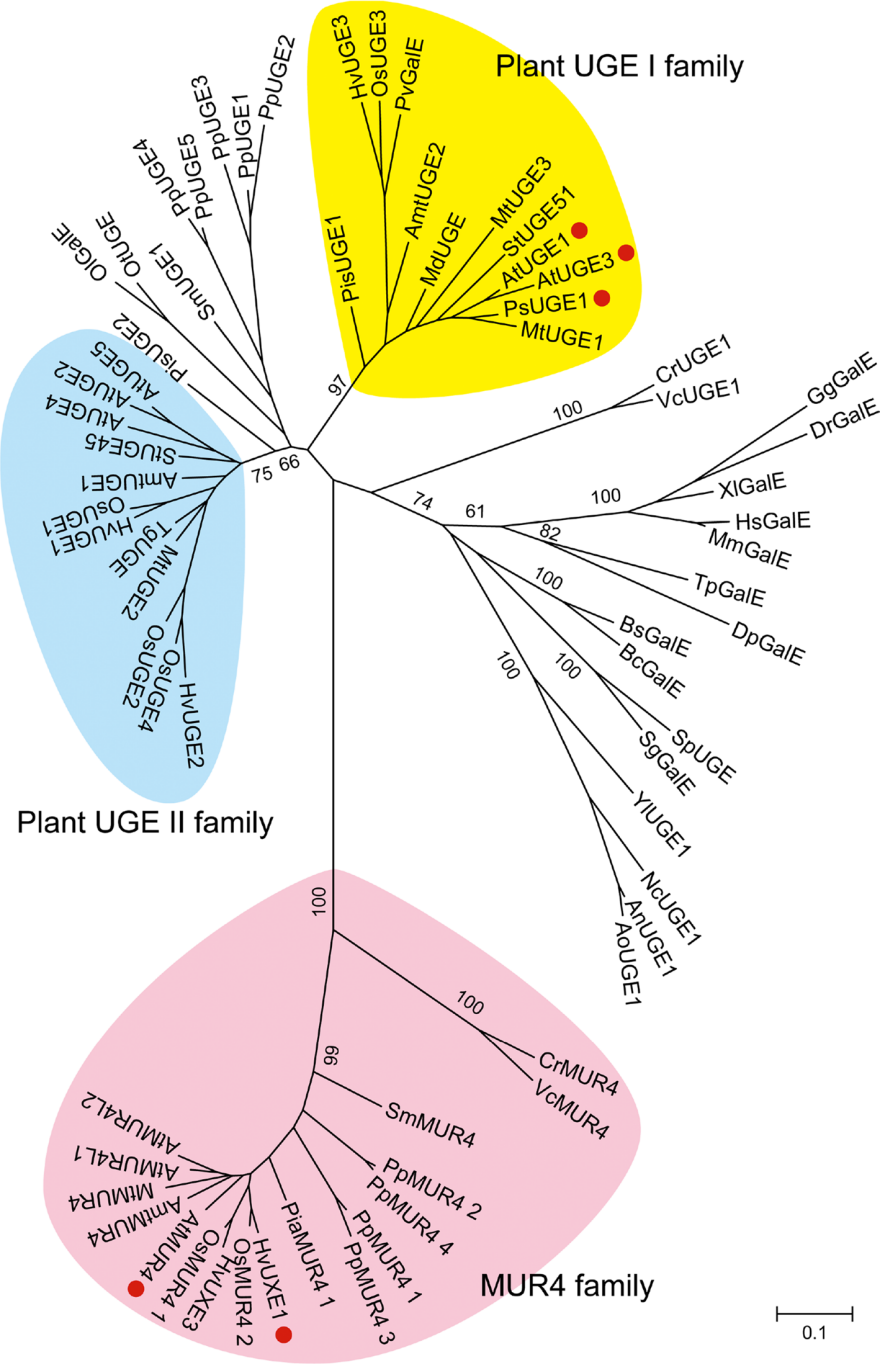
Relationships of MUR4 homologues and UGEs. The phylogenetic relationships of MUR4 homologues and UGEs were analyzed using MEGA software (version 6.0, Tamura et al. 2013). The *bar* indicates substitutions per site. *Red circles* indicate enzymes that have been shown to possess UXE activity. MUR4-related proteins were taken without their transmembrane domain, which was removed according to the prediction obtained from the TMHMM program (Krogh et al. 2001). Accession numbers for the sequences are listed in Supplemental Table 1

UGE is one of the most highly conserved enzymes and found not only in eukaryotes but also in prokaryotes. In mammals, it is called UDP-Gal 4-epimerase (GalE), as the enzyme catalyzes interconversion between UDP-Glc and UDP-Gal, which constitutes the Leloir pathway for the detoxification of Gal. Phylogenetic relationships suggest that angiosperm UGEs can be grouped into UGE I and II families (Fig. 4). In Arabidopsis, two out of five UGEs, UGE1 and UGE3 belong to the UGE I family and the other three UGEs, UGE2, UGE4, and UGE5 s to the UGE II family. Biochemical characterization using recombinant UGEs expressed in *Escherichia coli* showed that Arabidopsis UGE1 and UGE3 have UXE activity beside UDP-Glc 4-epimerase activity but UGE2, UGE4, and UGE5 s have none or only weak UXE activity (Kotake et al. 2009). Interestingly, the Norway spruce (*Picea sitchensis*, gymnosperm) genome includes a gene encoding UGE corresponding to Arabidopsis UGE1 and UGE3, whereas no UGE from *Physcomitrella patens* (moss) and *Selaginella moellendorffii* (spikemoss) was grouped into the UGE I family (Fig. 4). These facts lead to the hypothesis that the UGE I family with UXE activity recently evolved from UGE without UXE activity. To support this conjecture, it would be necessary to characterize other UGEs, particularly UGEs in gymnosperm and moss. In fact, only two MUR4-related proteins, Arabidopsis MUR4 and barley (*Hordeum vulgare*) UXE1 (Zhang et al. 2010), and three bifunctional UGEs, Arabidopsis UGE1 and UGE3 and pea (*Pisam sativum*) UGE1, have been shown to possess UXE activity so far (Fig. 4).

In sharp contrast with the plant UGE I family, close homologues to Arabidopsis MUR4 can be found not only in gymnosperms, ferns, and mosses, but also in green algae. Although the biochemical properties of MUR4 homologues in green algae have not been determined, the existence of l-Ara-containing glycoprotein and UAM catalyzing subsequent conversion of UDP-l-Ara*p* to UDP-l-Ara*f* strongly suggests their role as UXE (Bollig et al. 2007; Kotani et al. 2013). Together with algal homologues, MUR4 and MUR4 homologues in land plants form a subclade apart from plant the UGE I and II families, suggesting that MUR4 is a highly conserved old enzyme in plants.

## Degradation of l-Ara containing molecules

l-Ara-containing molecules undergo hydrolysis by glycoside hydrolases (GHs). The α-l-Ara*f* residues of pectic arabinan, arabinoxylan, and AGP are hydrolyzed by α-l-arabinofuranosidases belonging to the GH family (GHF) 3 and GHF 51 in the cell walls in land plants (Fig. 3). Many plant GHF 3 and GHF 51 α-l-arabinofuranosidases are bifunctional enzymes with β-xylosidase activity (Arsovski et al. 2009; Kotake et al. 2006; Lee et al. 2003; Minic et al. 2004; Tateishi et al. 2005). A native GHF 3 α-l-arabinofuranosidase/β-xylosidase purified from radish, RsAraf1, hydrolyzes pectic arabinan, type I AG, AGP (type II AG), and arabinoxylan showing broad substrate specificity toward α-l-Ara*f* residues (Hata et al. 1992). An Arabidopsis mutant with a defect in a GHF 51 α-l-arabinofuranosidase/β-xylosidase gene, *araf1*, exhibits accumulation of pectic arabinan in vascular tissues (Chávez Montes et al. 2008).

The enzyme hydrolyzing β-l-Ara*p* residues of AGP has not been identified, but candidate genes exist in land plants. Microbial GHF 27 β-l-arabinopyranosidases acting on β-l-Ara*p* residue of larch (*Larix laricin*a) type II AG have been reported (Ichinose et al. 2009; Salama et al. 2012). Based on the similarity of amino acid sequences, four genes in the genome of Arabidopsis are presumed to encode GHF 27 β-l-arabinopyranosidase or α-galactosidase. As the structure of β-l-Ara*p* resembles that of α-Gal (Fig. 1), it is not surprising that β-l-arabinopyranosidase and α-galactosidase exhibit quite similar three dimensional structures (Ichinose et al. 2009).

The hydrolysis of β-l-Ara*f* residues of arabino-oligosaccharides of extensin and CLE peptides has so far remained elusive. A bacterial β-l-arabinofuranosidase including a domain of unknown function (DUF) 1680 has been identified in *Bifidobacterium longum* (Fujita et al. 2014). Several proteins of Arabidopsis have this domain, but the similarity of amino acid sequences is low (identity at amino acid level, <15 %). It is necessary to examine whether these proteins act on the β-l-Ara*f* residues. No α-l-arabinopyranosidase acting on α-l-Ara*p* residues of RG-II is known at all. It is conceivable that to some extent, α-l-Ara*p* residues are hydrolyzed by GHF 35 β-galactosidase that widely exists in land plants, because α-l-Ara*p* and β-Gal are structurally similar.

## Recycling of free l**-**Ara released in the degradation

Free l-Ara released during the degradation and metabolism of l-Ara-containing molecules is recycled in the salvage pathway for the generation of nucleotide sugars. The enzymes constituting the salvage pathway are listed in Table 3. l-Ara*p* is first phosphorylated by l-arabinokinase1 (ARA1) and turned into l-Ara*p* 1-P (Fig. 3) (Dolezal and Cobbet 1991; Gy et al. 1998; Sherson et al. 1999). l-Ara*p* 1-P is then converted to UDP-l-Ara*p* by USP in the cytosol (Kotake et al. 2004, 2007; Litterer et al. 2006). This metabolic pathway probably functions as a third pathway for the generation of UDP-l-Ara*p* parallel to the dual de novo pathways occurring in the Golgi apparatus and cytosol (Fig. 3). It is interesting that two plant aldopentoses, l-Ara and Xyl, undergo different metabolism although they are C-4 epimer sugars of each other (Fig. 1). Free Xyl differs from l-Ara in that it is predicted to be converted to xylulose by Xyl isomerase (Maehara et al. 2013) and metabolized in the pentose phosphate pathway. The salvage pathway for free l-Ara is implicated in the detoxification of l-Ara: an Arabidopsis *ara1* mutant shows a severe growth defect in the presence of a high concentration of l-Ara (Dolezal and Cobbett 1991). Although no homozygous *usp* mutant has been analyzed—because USP is necessary for pollen development in Arabidopsis—, Geserick and Tenhaken (2013) have demonstrated the physiological importance of USP in vegetative tissue using *USP*-knock down (*kd*-*usp*) Arabidopsis. The *kd*-*usp* plant exhibited dwarf phenotype accumulating much free l-Ara and Xyl.

## Physiological importance of the l-Ara salvage pathway in pollen development

Observing the remarkable reduction of cell wall l-Ara in Arabidopsis *mur4* mutant (Burget and Reiter 1999; Burget et al. 2003), one is tempted to conclude that UDP-l-Ara*p* is mainly synthesized in the de novo pathway. However, several lines of evidence indicate the physiological importance of the salvage pathway for UDP-l-Ara*p* in developing pollens (Table 4). First, the rice l-arabinokinase named Collapsed Abnormal Pollen 1 (CAP1) has been shown to be necessary for normal development of pollens (Ueda et al. 2013), unfortunately, the effect of Arabidopsis *ara1* mutation on pollen development has not been studied. Second, the pollen development also appears to be influenced by lack of USP that catalyzes the subsequent conversion of l-Ara 1-P to UDP-l-Ara*p* as an Arabidopsis heterozygous *usp* mutant did not give any homozygous *usp* mutant (Kotake et al. 2007). In addition, collapsed pollens were observed in the anthers of heterozygous *usp* mutants and *kd*-*usp* plants (Geserick and Tenhaken 2013; Schnurr et al. 2006). As is the case in the vegetative tissues, UDP-l-Ara*p* generated in the salvage pathway is probably converted to UDP-l-Ara*f* by the action of UAM/RGPs in developing pollens. UAM/RGP was first identified as a factor necessary for the development of pollen in Arabidopsis (Drakakaki et al. 2006). Consistent with the phenotype of the rice *cap1* mutant and the Arabidopsis *usp* mutant, collapsed pollen grains were also observed in the anthers of homo/hetero *rgp1* *rgp2* double mutants (*rgp1*/*rgp1* *RGP2*/*rgp2* mutant) (Drakakaki et al. 2006). Furthermore, RNA-interference of the rice *UAM3* gene results in the formation of abnormal pollens lacking starch inside (Sumiyoshi et al. 2015). The importance of pectic arabinan for pollen development has been shown in the potato (Cankar et al. 2012), therefore it is highly probable that for pollen development the synthesis of a physiologically important portion of pectic arabinan depends on the salvage pathway.

**Table 4:** Mutants with defects in pollen development

Mutant name	Plant species	Enzyme activity	Phenotype	References
*ara1*	At^a^	l-Arabinokinase	Not determined^b^	Dolezal and Cobbet (1991)
*cap1*	Os^a^	l-Arabinokinase	Collapsed pollen	Ueda et al. (2013)
*usp*	At	UDP-sugar pyrophosphorylase	Collapsed pollen	Schnurr et al. (2006)
*rgp1/2*	At	UDP-l-Ara*p* mutase	Collapsed pollen	Drakakaki et al. (2006)
*uam3*	Os	UDP-l-Ara*p* mutase	Abnormal pollen	Sumiyoshi et al. (2015)

^a^At and Os indicate Arabidopsis and rice, respectively

^b^Phenotype in pollen development has not been studied

## Conclusion and future prospects

The plant-specific sugar l-Ara is generated as a form of UDP-l-Ara*p* through C-4 epimerization of UDP-Xyl in the de novo pathway. This reaction is catalyzed by MUR4 in the Golgi apparatus and by bifunctional UGE in the cytosol. However, the exact extent of the cytosolic contribution to the synthesis of l-Ara-containing molecules and the physiological role of this alternate pathway are not quite clear. l-Ara appears as α-l-Ara*f*, β-l-Ara*f*, α-l-Ara*p*, and β-l-Ara*p* residues in plants. Various GTs and GHs are involved in the synthesis and degradation of these residues, but many pieces of the puzzle, in particular enzymes dealing with β-l-Ara*f* and α-l-Ara*p* residues, remain to be identified. Reasons for slow progress on this question may include the difficulty in the preparation of l-Ara-containing molecules as substrates. Given the physiological importance of l-Ara*f*, it is not surprising that it is involved in various metabolic pathways. However, it is still unclear what significance in the evolution of plants the diversification of l-Ara use and the later emergence of a new pathway in the cytosol had. It would be of great interest to determine the relationship between other evolutionary events and the diversification of the l-Ara metabolism.

## Electronic supplementary material

Below is the link to the electronic supplementary material.

Supplementary material 1 (DOC 38 kb)
