# Correction to: Metabolism of l-arabinose in plants

**DOI:** 10.1007/s10265-018-1012-2

**Published:** 2018-02-21

**Authors:** Toshihisa Kotake, Yukiko Yamanashi, Chiemi Imaizumi, Yoichi Tsumuraya

**Affiliations:** Division of Life Science, Graduate School of Science and Engineering, Saitama University, 255 Shimo-okubo, Sakura-ku, Saitama, 338-8570 Japan

## Correction to: J Plant Res (2016) 129:781–792 10.1007/s10265-016-0834-z

The article “Metabolism of l-arabinose in plants”, written by “Toshihisa Kotake, Yukiko Yamanashi, Chiemi Imaizumi, Yoichi Tsumuraya”, was originally published Online First without open access. After publication in volume 129, issue 5, page 781–792 the Botanical Society of Japan decided to opt for Open Choice and to make the article an open access publication. Therefore, the copyright of the article has been changed to © The Author(s) 2018 and the article is forthwith distributed under the terms of the Creative Commons Attribution 4.0 International License (http://creativecommons.org/licenses/by/4.0/), which permits use, duplication, adaptation, distribution and reproduction in any medium or format, as long as you give appropriate credit to the original author(s) and the source, provide a link to the Creative Commons license, and indicate if changes were made.

