# Supplementary material for: Metabolism of l-arabinose in plants
Source: J Plant Res. 2016 May 24;129(5):781–92. doi: 10.1007/s10265-016-0834-z (PMC5897480; doi:10.1007/s10265-016-0834-z)
Supplement: Supplementary file 1 — Supplementary material 1 (DOC 38 kb) [file 10265_2016_834_MOESM1_ESM.doc]

**Electric supplementary materials**

**Title:**

Metabolism of L-arabinose in plants

**Authors:**

Toshihisa Kotake, Yukiko Yamanashi, Chiemi Imaizumi, Yoichi Tsumuraya

**Journal:**

Journal of Plant Research

**Corresponding author:**

Toshihisa Kotake

Division of Life Science, Graduate School of Science and Engineering, Saitama University, 255 Shimo-okubo, Sakura-ku, Saitama 338-8570, Japan

E-mail: kotake@mail.saitama-u.ac.jp

Tel: +81-(48)-858-3955

Fax: +81-(48)-858-3384

**Content:**

**Table S1**

**Table S1** List of MUR4s and UGEs analyzed in Fig. 4

Name Source organism Gene locus or Accession number

AmtMUR4 *Amborella trichopoda* XP_006845984.1

AmtUGE1 *A. trichopoda* XP_006849498.1

AmtUGE2 *A. trichopoda* XP_011628345.1

AnUGE1 *Aspergillus niger* EHA27834.1

AoUGE1 *Aspergillus oryzae* XP_001827449.1

AtUGE1 *Arabidopsis thaliana* AT1G12780.1

AtUGE2 *A. thaliana* AT4G23920.1

AtUGE3 *A. thaliana* AT1G63180.1

AtUGE4 *A. thaliana* AT1G64440.1

AtUGE5 *A. thaliana* AT4G10960.1

AtMUR4 *A. thaliana* AT1G30620.1

AtMUR4L1 *A. thaliana* AT4G20460.1

AtMUR4L2 *A. thaliana* AT5G44480.1

BcGalE *Bacillus cytotoxicus* WP_012096410.1

BsGalE *Bacillus subtilis*  AIY95202.1

CrMUR4 *Chlamydomonas reinhardtii* XP_001695212.1

CrUGE1 *C. reinhardtii* XP_001698706.1

DpGalE *Drosophila pseudoobscura* XP_001352806

DrGalE *Danio rerio* NP_001035389

GgGalE *Gallus gallus* XP_417833

HsGalE *Homo sapiens*  Q14376

HvUGE1 *Hordeum vulgare* AAX49504

HvUGE2 *H. vulgare* AAX49505

HvUGE3 *H. vulgare* AAX49503

HvUXE1 *H. vulgare* ABC67797.1

HvUXE3 *H. vulgare* ABC67799.1

MdUGE *Malus domestica*  BAF51705

MmGalE *Mus musculus*  NP_848476

MtMUR4 *Medicago truncatula* XP_013463086.1

MtUGE1 *M. truncatula* XP_013446997.1

MtUGE2 *M. truncatula* XP_003610981.1

MtUGE3 *M. truncatula* XP_013458702.1

NcGAL10 *Neurospora crassa* NP_957519.1

OlGalE *Ostreococcus lucimarinus* NC_009362.1

continued

OsMUR4_1 *Oryza sativa* Os08g0129700

OsMUR4_2 *O. sativa* Os07g0139400

OsUGE1 *O. sativa* Os05g0595100

OsUGE2 *O. sativa* Os08g0374800

OsUGE3 *O. sativa* Os09g0526700

OsUGE4 *O. sativa* Os09g0323000

OtMUR4 *Ostreococcus tauri* Ot09g01830

OtUGE *O. tauri* Ot08g01550

PiaMUR4_1 *Picea abies* MA_28209g0010

PisUGE1 *Picea sitchensis* ABR16202.1

PisUGE2 *P. sitchensis* ABR16828.1

PsUGE1 *Pisum sativum*  AB381885

PvGalE *Paspalum vaginatum* BAE92559.1

PpMUR4_1 *Physcomitrella patens* XP_001759327.1

PpMUR4_2 *P. patens* XP_001763148.1

PpMUR4_3 *P. patens* XP_001780353.1

PpMUR4_3 *P. patens* XP_001773632.1

PpUGE1 *P. patens* XP_001768301.1

PpUGE2 *P. patens* XP_001777464.1

PpUGE3 *P. patens* XP_001775163.1

PpUGE4 *P. patens* XP_001751529.1

PpUGE5 *P. patens* XP_001771084.1

SgUGE *Streptococcus gordonii* Q83WI1

SmMUR4 *Selaginella moellendorffii* XP_002990605.1

SmUGE1 *S. moellendorffii* XP_002963168.1

SpUGE *Streptococcus pneumoniae* WP_001156541.1

StUGE45 *Solanum tuberosum* AAP42567.1

StUGE51 *S. tuberosum* AAP97493.1

TgUGE *Toxoplasma gondii* O65781

TpGalE *Tribolium castaneum* XP_968616.1

VcMUR4 *Volvox carteri* XP_002947079.1

VcUGE1 *V. carteri* XP_002957530.1

XlGalE *Xenopus laevis* NP_001080902

YlUGE1 *Yarrowia lipolytica* XP_504440.1
